# The usefulness of global longitudinal peak strain and left atrial volume index in predicting atrial fibrillation in patients with ischemic stroke

**DOI:** 10.3389/fneur.2023.1287609

**Published:** 2024-01-05

**Authors:** Soo-Hyun Park, Yerim Kim, Minwoo Lee, Sang-Hwa Lee, Jong Seok Bae, Ju-Hun Lee, Tae Jung Kim, Sang-Bae Ko, Sang-Wuk Jeong, Dong-Eog Kim, Wi-Sun Ryu

**Affiliations:** ^1^Department of Neurology, Hallym University Kangdong Sacred Heart Hospital, Seoul, Republic of Korea; ^2^Department of Neurology, Hallym University Sacred Heart Hospital, Hallym Neurological Institute, Hallym University College of Medicine, Anyang, Republic of Korea; ^3^Department of Neurology, Chuncheon Sacred Heart Hospital, Hallym University College of Medicine, Chuncheon, Republic of Korea; ^4^Department of Neurology, Seoul National University Hospital, Seoul, Republic of Korea; ^5^Department of Neurology, Dongguk University Ilsan Hospital, Goyang, Republic of Korea

**Keywords:** atrial fibrillation, left atrial volume index, global longitudinal peak strain, embolic stroke of undetermined source, ischemic stroke, speckle-tracking imaging echocardiography

## Abstract

**Introduction:**

Detection of atrial fibrillation (AF) is crucial for preventing recurrence in patients with ischemic stroke. We aimed to examine whether the left atrial volume index (LAVI) and global longitudinal peak strain (GLPS) are associated with AF in patients with ischemic stroke.

**Methods:**

We prospectively analyzed 678 consecutive patients with ischemic stroke. LAVI and GLPS were assessed using three-dimensional transthoracic echocardiography with speckle-tracking imaging. Multiple logistic regression was used to evaluate the association of AF with LAVI and GLPS. To evaluate the predictive value of LAVI and GLPS for the presence of AF, we used optimism-corrected c-statistics calculated by 100 bootstrap repetitions and the net reclassification improvement (NRI).

**Results:**

The mean patient age was 68 ± 13 years (men, 60%). Patients with AF (18%) were a higher LAVI (41.7 ml/m^2^ vs. 74.9 ml/m^2^, *P* < 0.001) and a higher GLPS than those without AF (−14.0 vs. −17.3, *P* < 0.001). Among the 89 patients classified with embolic stroke of unknown source, the probable cardioembolic group had higher GLPS (*n*= 17, −14.6 vs. −18.6, respectively; *P*= 0.014) than the other groups (*n*= 72). Adding GLPS to age, hypertension, and the LAVI significantly improved the NRI, with an overall NRI improvement of 6.1% (*P*= 0.03).

**Discussion:**

The LAVI andGLPS with speckle-tracking imaging echocardiography may help identify patients with AF.

## 1 Introduction

Approximately 10–40% of patients with ischemic stroke are classified as having cryptogenic stroke despite thorough investigations (1, 2). Undetected atrial fibrillation (AF) is considered the major cause of cryptogenic stroke (3), leading to clinical trials evaluating the efficacy of anticoagulation drugs compared with antiplatelet agents in patients with embolic stroke of undetermined source. However, recent trials have failed to show the superiority of direct oral anticoagulant agents over antiplatelet agents in patients with embolic stroke of undetermined source (4, 5). In addition, a long-term follow-up study using an implantable cardiac monitor in patients with embolic stroke of undetermined source reported that the annual AF detection rate was only 12% (2, 6). Hence, targeting patients at risk of AF is necessary for efficient screening of AF in future clinical trials (7, 8).

Several clinical factors, including age and hypertension, are associated with AF. In particular, an enlarged left atrium has been consistently associated with the presence and future risk of AF (9–11). Speckle-tracking echocardiography evaluates the effect of left ventricular (LV) strain during the cardiac cycle on left atrium performance (12–14). Two studies have reported that LV strain is associated with the presence of AF, and these findings warrant further study (11, 15).

Speckle tracking echocardiography is a valuable technique to evaluate myocardial deformation and assess myocardial function (16). Among parameters of LV speckle tracking, the global peak longitudinal peak strain (GLPS) is the most widely investigated for systolic function (16). GLPS can detect compromised systolic function early despite complementing the LVEF (17). It might additionally assist as a sensitive marker of adverse events, including AF. Therefore, we sought to assess whether GLS predicts AF in stroke patients.

We hypothesized that left atrial (LA) volume and GLPS of the left ventricle were associated with AF. Furthermore, we evaluated the efficacy of GLPS in classifying patients at high risk of AF.

## 2 Materials and methods

### 2.1 Study population

This study is an observational and cross-sectional study. We prospectively enrolled 904 consecutive patients with ischemic stroke who had been admitted to our hospital within seven days after symptom onset between January 2014 and December 2016. Patients who had not undergone speckle-tracking echocardiography due to poor patient cooperation and severe neurological deficits were excluded (*n* = 220). In addition, six patients who did not have essential echocardiographic data, including LA volume and GLPS, were excluded. Finally, 678 patients were included in the analysis. All patients underwent standard evaluation, treatment, and rehabilitation in line with pre-specified guidelines for ischemic stroke (18). The Institutional Review Board of Dongguk University Ilsan Hospital approved this study. All patients or their legally authorized representatives provided written informed consent for participation in the study. The present study protocol was reviewed and approved by the Institutional Review Board of Dongguk University Ilsan Hospital (approval No. 2011-01-098). Informed consent was submitted by all subjects when they were enrolled.

### 2.2 Clinical data and stroke subtypes

We collected demographic data, prior medication history, laboratory data, and data on risk factors (hypertension, diabetes mellitus, hyperlipidemia, coronary artery disease, AF, and smoking history). Stroke subtypes were determined according to the consensus of experienced neurologists in the center, using a validated magnetic resonance imaging (MRI)-based algorithm, based on the Trial of Org 10172 in Acute Stroke Treatment (TOAST) criteria: large artery atherosclerosis, small vessel occlusion, cardioembolism, other determined, or undetermined stroke (19). Further, patients who had an undetermined stroke with a negative etiology (cryptogenic stroke) were categorized according to an evidence-based causative classification system (CSS) to differentiate probable cardioembolic stroke, large artery atherosclerosis, cardio-aortic embolism, small artery occlusion, other uncommon causes, and undetermined causes (19, 20). The CSS includes a category of probable cardioembolic stroke based on brain imaging, despite no evidence of a high-risk cardiac source of cerebral embolism (20–24).

### 2.3 Echocardiographic data

Three-dimensional (3D) transthoracic echocardiography was performed by an experienced cardiac sonographer using Vivid E9 Ultrasound (General Electric Medical Systems, Milwaukee, WI, USA) and a 4V-D ultrasound probe (General Electric Medical Systems, Milwaukee, WI, USA) following the standardized protocols recommended by the American Society of Echocardiography and European Association of Cardiovascular Imaging (25, 26). The echocardiographic variables were analyzed offline within the EchoPac platform (GE Medical Systems, Milwaukee, WI, USA). The time interval between the QRS onset on the electrocardiogram and aortic and mitral valve opening and closure was measured using pulsed-wave Doppler from the LV outflow and inflow. During the measurements, three cycles of sinus rhythm and five cycles of atrial fibrillation were averaged. The parameters (LV volume, LV ejection fraction [LVEF], peak early velocity [E], peak early diastolic velocity [e'] of the lateral and septal mitral annulus, E/e' ratio, LV end-diastolic diameter [LVED], LV end-systolic diameter [LVESD], and LV mass) were evaluated in accordance with recommendations using the modified biplane Simpson's rule and calculated using the 3D data set, which was validated by a specialized independent cardiologist who was blinded to the clinical data (27, 28). LA volume was measured using a biplane Simpson's method in apical four- and two-chamber views and indexed to the body surface area (ml/m^2^) (24, 29–31).

### 2.4 Speckle-tracking imaging echocardiography

We used 3D speckle-tracking echocardiography to assess LV GLPS and to evaluate the endocardium and epicardium for myocardial deformation using 3D wall motion tracking software (General Electric Medical Systems, Milwaukee, WI, USA) automatically, according to the guidelines of the American Society of Echocardiography and European Association of Cardiovascular Imaging (25, 26, 32). The cardiac cycle was marked off, indicating the QRS onset, and the QRS complex was used as the first reference frame. At least two cardiac cycles were recorded and averaged, and the frame rates were set to 60–80 frames/s. LV GLPS was measured using speckle-tracking imaging echocardiography from four-chamber, two-chamber, and apical longitudinal long-axis view with a breath-hold during the echocardiogram recording. An inspector could specify the area of interest. After 16, 17, or 18 segmental tracking analyses and a final manual analysis of the area of interest or automatically designed area, longitudinal strain curves were constructed using the software for each ventricular segment. According to each segment, LV GLPS was measured to average the negative peak of longitudinal strain (movement of the base toward the apex), with more negative values expressing better systolic function.

### 2.5 Statistical analysis

Data are presented as mean (standard deviation [SD]), median (interquartile range [IQR]), and number (percentage), as appropriate. A Kolmogorov-Smirnov test was used to assess the normality distribution of continuous variables. Parametric data were assessed using either the student's *t*-test or analysis of variance (ANOVA) for continuous variables. For non-parametric data, either the Wilcoxon rank-sum or Kruskal-Wallis tests were used. χ^2^ or Fisher's exact tests examined the association between categorical variables. Multiple logistic regression analysis was used to examine the association between AF and the variables while adjusting for covariates. Variables with a *P*-value < 0.1 in the univariate analysis were entered into the multivariable model. Binary logistic regression analysis was used to investigate the effect of the left atrial volume index (LAVI) and GLPS in CSS-categorized patients with cardioembolic stroke. To evaluate the predictability of the LAVI and GLPS for the presence of AF, we used optimism-corrected c-statistics calculated using 100 bootstrap repetitions and net reclassification improvement (NRI) (31, 32). The c-statistic is a measure of stratification, and the NRI specifies the amount of correct reclassification of an estimated AF presence (8). The NRI estimates were based on reclassification tables classifying patients into prespecified AF risk categories, namely, low (10%), intermediate (10% to 30%), and high (≥30%). The receiver operating characteristic (ROC) curve was used to examine the discriminatory capacity of LAVI and GLPS for the prediction of AF. ROC analyses were expressed as curve plots and calculated area under the curve (AUC) with confidence interval (CI). Data were analyzed using STATA (version 14.0; STATA Corp., College Station, Texas, USA), R 3.5.3, and GraphPad Prism (Version 9.0, GraphPad Software, San Diego, CA, USA) software, and two-tailed *P*-values < 0.05 were considered statistically significant.

## 3 Results

The mean patient age was 68 (SD, 13) years, 407 (60%) patients were men, and 122 (18%) patients had AF. Patients with AF were older (75 vs. 66 years, *P* < 0.001), high NIHSS (5 vs. 4, *P* < 0.001), and had a more frequent prior history of stroke than those without AF (25% vs. 17%, respectively, *P* = 0.03; Table 1). Echocardiography data indicated that those with AF had a lower LVEF (58% vs. 62%, *P* < 0.001) and a higher LAVI (75 ml/m^2^ vs. 42 ml/m^2^, *P* < 0.001) and E/e' (14.2 vs. 12.3, *P* < 0.001) than those without AF. In addition, GLPS (−14.0 vs. −17.3, *P* < 0.001) was higher in patients with AF than those without AF.

**Table 1:** Baseline characteristics stratified according to the presence of atrial fibrillation.

	**Atrial fibrillation**		***P-*value**
	**No (*****n** =* **556)**	**Yes (*****n** =* **122)**	
Age, mean ± SD (years)	66.4 ± 13.2	75.2 ± 10.1	<0.001
Male, *n* (%)	338 (68.0)	69 (56.6)	0.390
Hypertension, *n* (%)	437 (78.6)	99 (81.2)	0.530
Diabetes, *n* (%)	257 (46.2)	43 (35.3)	0.027
Hyperlipidemia, *n* (%)	311 (55.9)	66 (54.1)	0.710
Smoking, *n* (%)	189 (34.0)	24 (19.7)	0.002
Coronary artery disease, *n* (%)	34 (6.1)	13 (10.7%)	0.070
Previous stroke history, *n* (%)	95 (17.1)	31 (25.4)	0.030
Stroke subtype, *n* (%)			<0.001
Large artery atherosclerosis	239 (43.0)	0	
Small vessel occlusion	170 (30.6)	0	
Cardioembolism	11 (2.0)	87 (71.3)	
Undetermined		35 (28.7)	
Other determined	14 (2.5)	0	
Admission NIHSS, median (IQR)	4 (2–5)	5 (3–12)	<0.001
Ejection fraction, mean ± SD (%)	62.4 ± 6.4	57.8 ± 9.0	<0.001
Left atrial volume index, mean ± SD	41.7 ± 11.0	74.9 ± 33.9	<0.001
E/e', mean ± SD	12.3 ± 4.7	14.2 ± 5.6	<0.001
GLPS, mean ± SD (%)	−17.2 ± 4.9	−14.1 ± 5.2	<0.001

E/e', peak early velocity (E)/peak early diastolic velocity (e') of the lateral and septal mitral annulus; GLPS, global longitudinal peak strain; NIHSS, national institutes of health stroke scale; IQR, interquartile range; SD, standard deviation.

Multivariable logistic regression analysis showed that age (per year, adjusted odds ratio [OR] 1.05, 95% confidence interval [CI] 1.02–1.08) and hypertension (OR 0.44, 95% CI 0.21–0.93) were independently associated with the presence of AF (Table 2). In addition, GLPS (per 1% increase, OR 1.07, 95% CI 1.00–1.12) and the LAVI (OR 1.11, 95% CI 1.08–1.13) were also associated with AF.

**Table 2:** Multivariable logistic regression for the presence of atrial fibrillation.

	**Odds ratio (95% confidence interval)**	***P* value**
Age	1.05 (1.02–1.08)	0.003
Male	1.80 (0.91–3.58)	0.093
Hypertension	0.44 (0.21–0.93)	0.031
Diabetes	0.87 (0.47–1.61)	0.670
Hyperlipidemia	1.47 (0.80–2.71)	0.220
Smoking	0.56 (0.26–1.21)	0.140
Coronary artery disease	0.75 (0.26–2.18)	0.590
Ejection fraction	0.95 (0.92–0.99)	0.014
GLPS	1.07 (1.00–1.12)	0.023
E/e'	0.96 (0.90–1.03)	0.250
Left atrial volume index	1.11 (1.08–1.13)	<0.001

E/e', peak early velocity (E)/peak early diastolic velocity (e') of the lateral and septal mitral annulus; GLPS, global longitudinal peak strain.

**Figure 1:**
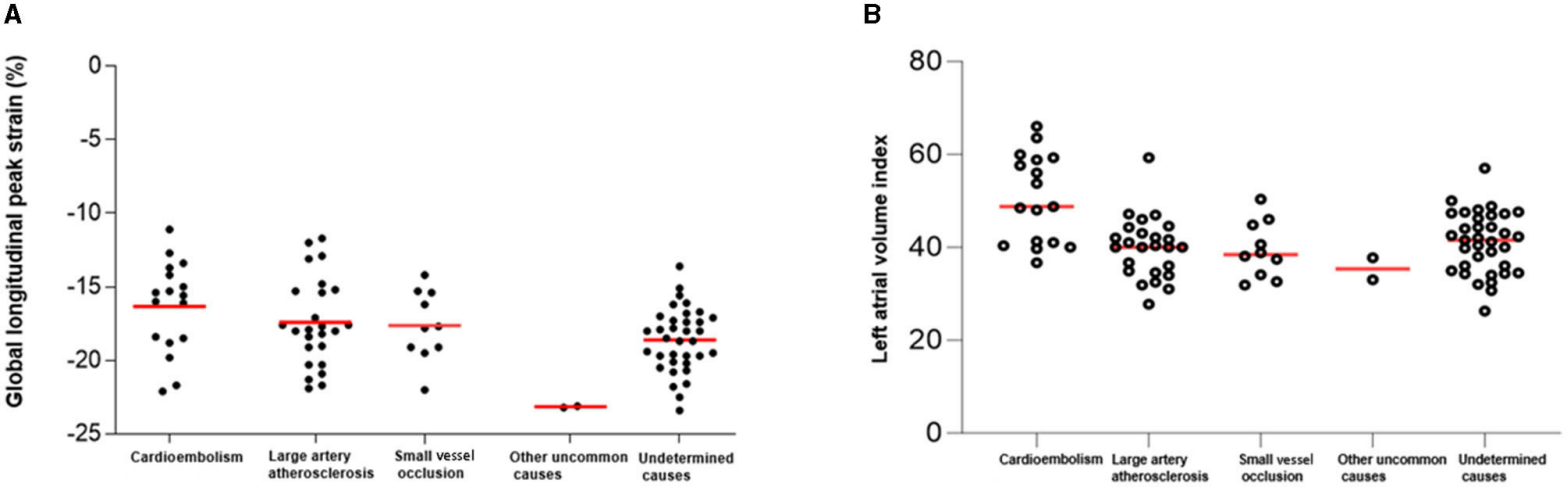
Global longitudinal peak strain (GLPS) and left atrial volume index (LAVI) according to causative classification system (CCS) subtype. According to the CCS subtype, the GLPS value **(A)** and LAVI **(B)** were the highest for cardioembolism.

**Figure 2:**
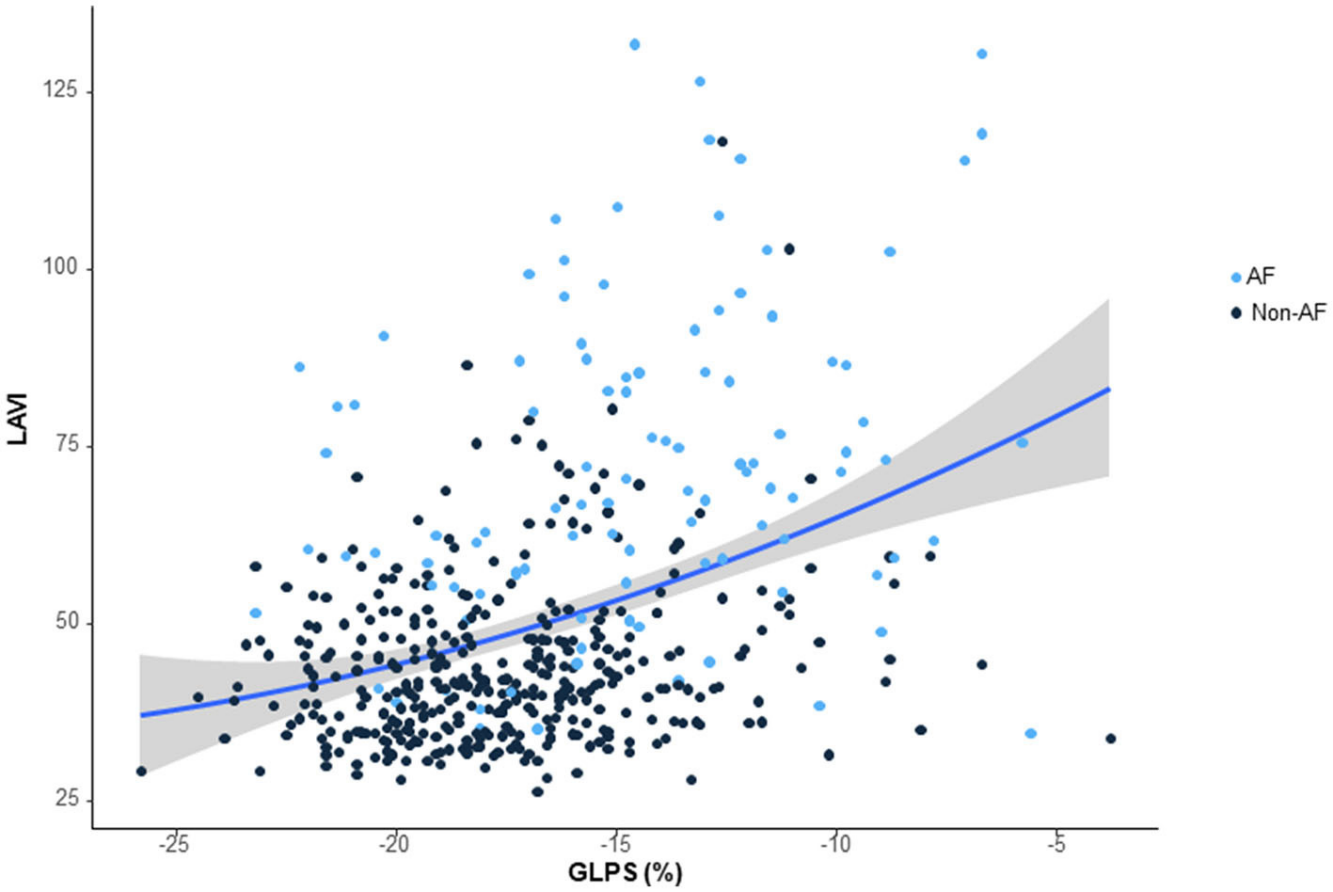
The relationship between global longitudinal peak strain (GLPS) and left atrial volume index (LAVI). The scatterplot shows the relationship between LAVI and GLPS. LAVI = 84.06 + 2 X LPS, R^2^ = 0.146; *P* < 0.001.

Of 89 patients who had been categorized as having an undetermined stroke with a negative etiology, 17 (19%) patients were reclassified as having probable cardio-aortic embolism (CE), 25 with large artery atherosclerosis, 10 with small vessel occlusion, 2 with other uncommon causes, and 35 with undetermined causes. In particular, GLPS and LAVI were higher in patients with CE than in patients with other causes (Figures 1A, B, *P* < 0.001). In addition, there was a significant correlation between the LAVI and GLPS in patients with CE (*R*^2^ = 0.146, *P* < 0.001; Figure 2).

When we dichotomized the patients into two groups, namely, the CSS CE group and CSS non-CE group, no statistically significant differences in terms of age (mean 67 vs. 69 years, respectively; *P* = 0.44) and prevalence of hypertension (94% vs. 78%, respectively; *P* = 0.11) was observed between the two groups. However, GLPS (−16.3 vs. −18.2, respectively; *P* = 0.014) and LAVI (45 vs. 41 ml/m^2^, respectively, *P* = 0.068) were both found to be higher when comparing patients in the CSS CE group with those in the CSS non-CE group.

**Figure 3:**
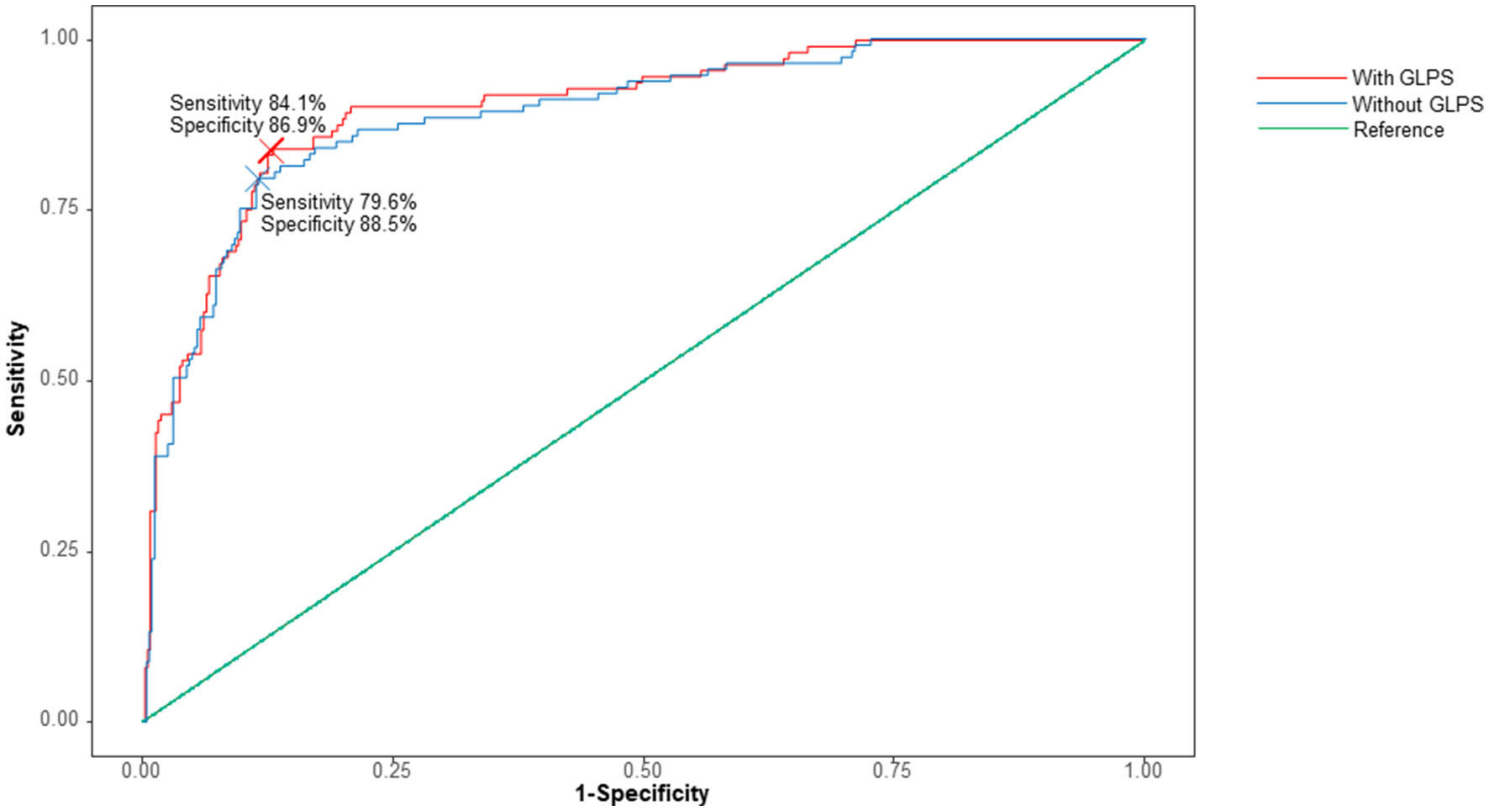
Receiver operating curve analysis with or without global longitudinal peak strain (GLPS). The baseline model includes age, hypertension, and left atrial volume index. The C-index for the model without GLPS (red line) was 0.8938 (95% CI 0.8594–0.9281). The C-index for the model with GLPS (blue line) was 0.9039 (95% CI 0.8717–0.9361, *P* = 0.028).

A receiver operating characteristic curve showed that the addition of GLPS to the baseline model for AF that included age, hypertension, and LAVI significantly increased the area under the receiver operating curve (*P* = 0.028, Figure 3). The C-index for the baseline AF model based on age, hypertension, and LAVI was 0.892 (0.857–0.928, *P* < 0.001). Adding GLPS to this model improved the C-index to 0.902 (0.869–0.935, *P* < 0.001). Although there was a slight difference, it improved the result with the addition of GLPS statistically significantly.

Table 3 shows the patients' classification into categories of predicted risk (<10%, 10– <30%, and ≥30%) in terms of age, hypertension, and LAVI and when they were combined with GLPS. Of the 122 patients with AF, seven were correctly reclassified into a higher-risk category, and two were reclassified into a lower-risk category. Of 556 patients without AF, 35 were correctly reclassified into a higher-risk category, and 24 were reclassified into a lower-risk category. The overall NRI was 6.1% (*P* = 0.03), and the Integrated Discrimination Index (IDI) was 0.011 (*P* = 0.039).

**Table 3:** Net reclassification improvement based on global longitudinal peak strain (GLPS).

**Patients with atrial fibrillation**				
**Without GLPS** ^*^	**With GLPS**			
	<**10%**	**10–30%**	≥**30%**	**Total**
<10%, *n*	10	4^‡^	-	14
10–30%, *n*	1^§^	16	3^‡^	20
≥30%, *n*	-	1^§^	87	88
Total, *n*	11	21	90	122
**Patients without atrial fibrillation**				
**Without GLPS** ^*^	**With GLPS**			
	<**10%**	**10–30%**	≥**30%**	**Total**
<10%, *n*	364	18^§^	1^§^	383
10–30%, *n*	26^‡^	103	5^§^	134
≥30%, *n*	-	9^‡^	30	39
Total, *n*	390	130	36	556

* Multivariable model including age, hypertension, and left atrial volume index.

‡ The number of patients who were correctly reclassified according to the model with GLPS.

§ The number of patients who were incorrectly reclassified according to the model with GLPS.

GLPS, global longitudinal peak strain.

## 4 Discussion

This study showed that the LAVI and GLPS with speckle-tracking imaging echocardiography could effectively discriminate between patients with AF and ischemic stroke. To the best of our knowledge, this study is the first to report GLPS and LAVI's clinical implications in the risk stratification of AF in patients with ischemic stroke. In addition, abnormal GLPS and LAVI facilitate risk prediction of AF in patients with suspected cardioembolic stroke and enhance the possibility of using routine clinical examination findings to determine the mechanism of ischemic stroke (33–36).

Acute stroke guidelines recommend that echocardiography, transthoracic echocardiography (TTE), or transesophageal echocardiography (TEE) are routine diagnostic methods for acute stroke (18, 37–39). There is controversy about performing TEE when finding sources of cardioembolic stroke. Because TEE strain assessment is superior to TTE strain, the correlation between TTE and TEE strain needs to be clarified, as evidenced by the conflicting reports. TEE can be used to detect embolic cardiac sources, including the aortic arch, left atrium, and atrial septum, as it is superior for evaluation to TTE (37). It can evaluate patent foramen ovale (PFO), which may recommend closure in younger patients with cryptogenic stroke (40–42). However, even TEE findings are often not well reflected or changed treatment with therapeutic decision-making. TEE is a relatively invasive method, more resource-intensive and expensive than TTE, which further adds to the expense (43). TTE has the advantage of being used widely, handily, timely, and portable, with a lower risk of procedural complications than TEE (44). To evaluate the LV, 3D TTE is equally effective as 3D TEE (45, 46). Therefore, we chose and used the 3D TTE for this study.

LA volume represents the end of the LV systolic contraction. LV dysfunction is related to reducing passive LA emptying during the cardiac cycle, leading to high atrial pressure during the LA diastolic phase (34, 47). Therefore, enlarged LA volume can result from hypertension, LV dysfunction, LV hypertrophy, and increased LA filling pressure (48). LA volume is considered a predictable marker of cardiovascular risk. Increased LA pressure stretches the atrial structures and may potentiate LA cellular remodeling, resulting in AF (10, 48). Previous studies have also reported that LA enlargement is related to the high prevalence of ischemic stroke and AF (7, 9, 15, 30, 49). In addition, increased LA volumes are associated with silent cerebrovascular lesions due to undetected AF (7, 36). In particular, cryptogenic stroke in patients with LA enlargement is more closely linked to AF than in those without LA enlargement (50–55).

There are various methods for assessing LA volumes (47). An increased LAVI is significantly associated with cardioembolic stroke and recurrent stroke in patients with AF (48, 53, 54). In line with previous reports, we found that the LAVI was an essential predictor of AF in patients with ischemic stroke, with a 1 mL/m^2^ increase associated with a 10% increase in the risk of AF. This key finding provides critical evidence concerning the risk of cardioembolic stroke and the need for additional evaluations for cryptogenic stroke (10, 55–57). Standardization and consistent use of the LAVI may help detect AF effectively due to LAVI assessment reproducibility (47).

Recent advances in imaging to non-invasively quantify LV function (58, 59) 3D with speckle tracking imaging echocardiography allow an objective evaluation of global and regional myocardial function. Reasonable spatial and temporal resolutions of 3D data are available to estimate exact myocardial motion. In contrast to the previous echocardiography, speckle-tracking imaging echocardiography has the advantage of evaluating an accurate presentation of active and passive myocardial motion (60, 61). If there is a deformed part of the systolic function, the longitudinal peak strain (GLPS) value may change (62).

GLPS detects LV functions more sensitively than LVEF as it assesses myocardial dysfunction (48, 63, 64). One study found abnormal GLPS values despite preserving the LVEF (65). Another study showed that GLPS effectively detected LA remodeling and independent predictors of AF. Because hemodynamic LA function strongly correlates with LV filling, assessing LA kinetics could provide practical information regarding the degree of ventricular dysfunction (65, 66).

In our study, as LAVI and GLPS are correlated with AF, we also showed that patients with cardioembolic stroke had significantly higher GLPS and LAVI values than other stroke mechanisms. Based on our results of increased NRI and C-index, GLPS may improve the detection of AF when combined with LAVI (67). Moreover, GLPS showed a stronger association with the LAVI, and these two variables can be used to predict AF. These findings have potential clinical implications in identifying other echocardiographic markers for assessing patients at high risk of stroke due to a cardioembolic source.

One strength of our study is the large sample size consisting of patients who had undergone speckle-tracking imaging echocardiography. A wide range of acute ischemic stroke profiles were observed in our study population. CCS classification was used in addition to stroke category classification. Moreover, advanced cardiologic parameters were linked to stroke subtypes, and our study results can assist in understanding the stroke mechanisms. However, this study had several limitations. First, the study sample included many Korean patients from a single center, which might limit the generalization of our results to populations with a different demographic distribution. Second, GLPS reflects intrinsic LV function. However, factors affecting extrinsic cardiac performance, such as preload, afterload, and medications, may interfere with cardiac function. Therefore, additional evaluation of GLPS after the acute period of ischemic stroke may need to be undertaken and compared with the GLPS in the acute phase. Third, the accuracy of GLPS may be affected by AF. Although our study measurements were averaged from five cardiac cycles in AF as proposed in the guidelines, the LV variable cycle with selected index beats in AF can be inaccurate. Fourth, we did not evaluate stroke patients with TTE. As we previously described, TEE has various advantages. However, because 3D TTE is known to be as effective as 3D TEE, our study was designed to ensure that it is convenient to use in actual clinical practice. Further research will be conducted using TEE and TTE. Finally, we conducted multivariate analyses adjusted for variable risk factors for acute ischemic stroke. However, unmeasured factors may have been involved in our study.

This study suggests the possible application of GLPS in clinical practice. Our study findings show that the LAVI and GLPS were significantly associated with the risk of cardioembolic stroke. In particular, the LAVI had a higher correlation with GLPS, and these results can help improve AF (21). GLPS may be used as an additional parameter for evaluating the mechanism of ischemic stroke. Further studies are recommended to determine the correlation between ischemic stroke volume or patterns and GLPS using the brain MRI, which represents the burden of acute ischemic stroke.

Our study is the first prospective study to show the potential clinical implications of the LAVI and GLPS for evaluating patients with suspected AF. Using speckle-tracking imaging echocardiography to assess the correlation between GLPS and LAVI is an effective method for identifying novel markers for classifying ischemic stroke.

## Data availability statement

The datasets generated for this study are available on request to the corresponding author.

## Author contributions

S-HP: Conceptualization, Investigation, Methodology, Writing – original draft, Writing – review & editing. YK: Funding acquisition, Project administration, Writing – review & editing. ML: Methodology, Writing – review & editing. S-HL: Software, Writing – review & editing. JB: Investigation, Supervision, Writing – review & editing. J-HL: Methodology, Writing – review & editing. TK: Supervision, Validation, Writing – review & editing. S-BK: Software, Validation, Writing – review & editing. S-WJ: Investigation, Methodology, Writing – review & editing. D-EK: Methodology, Supervision, Writing – review & editing. W-SR: Conceptualization, Data curation, Project administration, Writing – original draft, Writing – review & editing.
